# Novel tissue biomarker candidates to predict both deep venous thrombosis and healing outcome after Achilles tendon rupture

**DOI:** 10.1038/s41598-025-91511-0

**Published:** 2025-03-01

**Authors:** Annukka Saarensilta, Junyu Chen, Stefan Markus Reitzner, David A. Hart, Aisha S. Ahmed, Paul W. Ackermann

**Affiliations:** 1 Department of Molecular Medicine and Surgery, Karolinska Institutet, Stockholm, Sweden; 2 Department of Orthopaedic Surgery, West China Hosptial of Sichuan University, No.37 Guoxue Lane, Chengdu, 610041 Sichuan P. R. China; 3 Department of Physiology & Pharmacology, Karolinska Institutet, Stockholm, Sweden; 4 Department of Women’s and Children’s Health, Karolinska Institutet, Stockholm, Sweden; 5 Department of Surgery, Faculty of Kinesiology and the McCaig Institute for Bone & Joint Health, University of Calgary, Calgary, AB Canada; 6 Department of Trauma, Acute Surgery and Orthopaedics, Karolinska University Hospital, Stockholm, Sweden; 7 Department of Molecular Medicine and Surgery, K1 MMK Orthopaedics, Stockholm, 17176 Sweden

**Keywords:** Achilles tendon, Rupture, Biomarkers, Venous thrombosis, Heat-Shock response, Proteomics, Biochemistry, Cell biology, Biomarkers, Diseases, Health care, Medical research, Molecular medicine

## Abstract

Deep venous thrombosis (DVT) and poor long-term patient outcomes frequently occur in patients with Achilles tendon rupture (ATR). Biomarkers for DVT and their possible relationship to long-term healing outcomes remain unexplored. To identify DVT biomarkers from proteomic profiles during the inflammatory and proliferative healing stages and assess their associations with one-year healing outcomes after surgical repair of ATR. A cohort of 53 patients undergoing standardized ATR repair from previous clinical trials was investigated. Intraoperative inflammatory-stage tendon biopsies were obtained from 40 patients, and tendon microdialysates from 28 patients were collected two weeks later during the proliferative stage. Liquid chromatography-tandem mass spectrometry proteomic profiles were linked to DVT status at two weeks post-surgery using ultrasonography screening and to patient-reported outcomes at one-year post-surgery. Six candidate DVT biomarkers were identified from tendon biopsies, whereof four (ABI3BP, IGKV2-40/IGKV2D-40, PCYOX1, STIP1) were associated with one-year healing outcomes. In tendon microdialysates, 43 candidate DVT biomarkers were identified, but none were associated with healing outcomes. Bioinformatic analysis revealed pathways related to heat shock response, platelet signaling, collagen and extracellular matrix metabolism, and immunoglobulins. The results support shared inflammatory-stage protein pathways in regulating venous thrombosis and reported healing outcomes, where elements of individual hypoxic tolerance and platelet signaling emerge as potential key links.

## Introduction

Deep venous thrombosis (DVT) and unsatisfactory healing outcomes are common clinical problems after Achilles tendon rupture (ATR)^1–3^. DVT, which has a prevalence up to 50% during lower-leg immobillization after ATR^1,2^, can progress to pulmonary embolisms - a potentially lethal condition^4^ - or post-thrombotic syndrome^5^. Moreover, evidence suggests that patients who develop a DVT may experience impaired lower limb function one year after ATR^6–8^, indicating a possible link between early tendon healing events and subsequent development of venous thrombosis. The identification of shared biomarkers for venous thrombosis and healing outcomes remains unexplored, but such biomarkers could provide valuable insights into molecular mechanisms, improve prognostic tools, and facilitate the development of targeted therapies to enhance tissue healing and reduce DVT risk.

Dense connective tissues such as the Achilles tendon heal with varying degrees of fibrosis and other structural abnormalities^9,10^, leading to repair rather than true regeneration. Consequently, the optimal properties of a healthy tendon are never fully regained^11^. This suboptimal healing may be partly attributable to the slow metabolism of tendon tissues, although temporary neovascularization occurs early in the healing process to supply oxygen and nutrients^10^. Individual healing capacity is influenced by genetic and acquired factors, including immobilization, which is associated with impaired blood circulation and downregulation of healing-related genes^12–14^. Hypoxia, resulting from poor circulation, disrupts protein homeostasis, and physiological systems have evolved via evolution to counteract these negative effects^15^. However, hypoxia is a known risk factor for both thrombosis^16,17^ and poor healing^18,19^. A common factor linking healing disturbances and thrombosis development may be inter-individual differences in the tolerance to local hypoxia or variations in protein patways related to haemostasis, inflammation and collagen metabolism. Additionally, venous stasis following DVT may exacerbate poor healing outcomes. Given this complexity, advanced methodologies are required to decipher the molecular processes underlying these risks.

Modern mass-spectrometry-based proteomics provide means for a powerful, large-scale, non-biased approach for tissue profiling^20^. When combined with clinical data, this technology can identify biomarkers that provide improved understanding of the complex processes of thrombosis and healing under immobilization. Whether variations in protein profiles during early tendon healing are associated with the risk of developing DVT and long-term patient outcomes has yet to be determined.

The aim of the present study was to use proteomic profiling of early stages of ATR healing, with tissue biopsies as representative of the inflammatory healing stage and microdialysates acquired during the proliferative healing stage, to identify protein biomarkers for DVT in the local environment at two weeks post-surgery and their relationship to reported healing outcomes one-year post-surgery. It was hypothesized that a quantitative mass-spectrometry approach would uncover novel potential biomarkers for the risk of DVT and poor patient-reported outcome reflecting healing outcome, and their potential relationships.

## Materials and methods

### Study population

This is a cohort study based on data collected in prior randomized controlled trials (RCT) comparing different postoperative treatment protocols for ATR in Stockholm, Sweden, between 2011 and 2014 ^1,2^. Ethical permits were obtained from the regional ethical review board in Stockholm (Dnr: 2013-1791-31-3, 2009-2079-31-2). All the participants received oral and written information and provided written informed consent. All methods were performed in accordance with the relevant guidelines and regulations. All research was performed in accordance with the Declaration of Helsinki.

Similar inclusion and exclusion criteria were applied in both of the RCTs^1,21^. Inclusion criteria were unilateral acute ATR, age of 18–75 years, and surgery performed within 7 days of injury. Exclusion criteria were anticoagulant therapy, known kidney failure, heart failure with pitting oedema, thrombophlebitis, any thromboembolic event during the previous three months, other surgery during the previous month, known malignancy, hemophilia, pregnancy, unwillingness to participate, inability to follow instructions or to give informed consent or follow-up at another hospital.

**Fig. 1:**
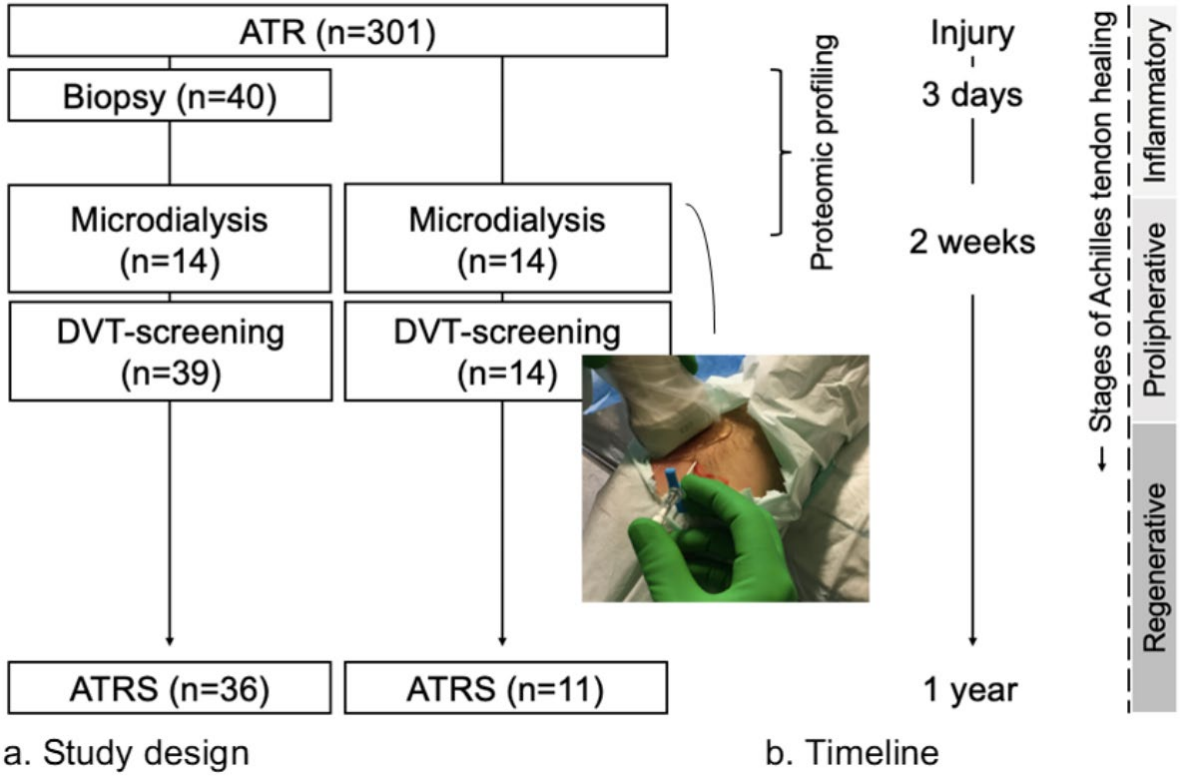
Study design and timeline. ATR = Achilles tendon rupture, ATRS = Achilles Tendon Rupture Score, DVT = Deep venous thrombosis. Microdialysis and DVT-screening were performed the same day.

Tendon biopsies and microdialysates were collected from voluntary patients after obtaining informed consent (Fig. 1). Forty biopsies were selected in a random manner by study personnel, as it was not feasible to analyze all biopsies with mass spectrometry due to financial constraints. Biopsies were selected from twenty patients with ATRS ≤ 75 and twenty patients with ATRS ≥ 85 (ATRS > 80 defined as a good outcome). DVT-screening status at two weeks was not available for one of the patients. Microdialysates were collected from fourteen of the forty patients on day of DVT-screening at two weeks after surgery. Microdialysates were also available from an additional fourteen patients.

### Surgical procedure and Achilles tendon biopsies

Standardized surgical ATR repair was performed on all study patients (Fig. 1.). The surgical procedure has been described previously^1,21^. Tendon biopsies were collected during surgery according to the study protocol. One 10 mm biopsy was collected at the rupture site.

### Microdialysis of Achilles tendon

To obtain fluid from the repaired injury site at two weeks post-repair, an introducer followed by a catheter (CMA 71; CMA Microdialysis AB; 100 kDa molecular cutoff, 0.5 mm outer diameter; 30 mm in length) with a semipermeable membrane was positioned in the peritendinous space of the Achilles tendon with ultrasound guidance under sterile conditions (Fig. 1.)^22^. The catheters were perfused with microdialysis fluid (Macrodex^®^) with a pump (CMA 107; CMA Microdialysis) speed of 1.0 µL/min^22^. The fluid was collected in four vials (Microvial, CMA Microanalysis AB) at each side for two hours (i.e., vials were changed at 30 min intervals)^22^. The procedure was completed by extracting the catheters and applying an adhesive bandage. The vials were stored in a freezer at -80 °C.

### Postoperative treatments

In the RCTs, after surgery all patients were randomly allocated for different treatments for six weeks^1,2^. The first group received standard treatment; a plaster cast in 30° equinus-position and unloading for two weeks followed by an orthosis (Aircast^®^Air Select™ Elite, DJO)^1,2^. The second group was treated with immediate orthosis and free loading combined with intermittent pneumatic compression (IPC) treatment for calves during the first two weeks^1^. The third group was treated with early functional mobilization (EFM) in an ankle mobile orthosis (VACOPed^®^, OPED)^2^.

### Deep venous thrombosis (DVT) screening

DVT-screenings were conducted at two weeks postoperatively^1,2^. Compression duplex ultrasonography (Philips CX 50 ultrasound machine, Philips Medical Systems). The diagnostic criteria used for DVT detection have been described previously^23^. Briefly, all deep proximal and distal veins including muscle veins and vena saphena magna in the injured leg were assessed by a trained nurse or an experienced ultrasonogapher^2^. Patients with DVT were treated with low-molecular-weight heparin according to the hospital protocol^1,2^.

### Registration of patient-reported outcome

To evalute healing outcome, patient-reported outcome was assessed at one-year postoperatively using the validated Achilles tendon Total Rupture Score (ATRS)^1,2,24^. The ATRS consists of 10 questions, each question provides a 0–10 value, and a score of 100 indicates full recovery^25^. An ATRS > 80 is considered a good patient reported outcome^6,7^.

### LC-Mass-spectrometry (MS)

Liquid chromatography-tandem mass spectrometry (LC-MS/MS) was used to create complete proteomic profiles of tendon biopsy extracts and microdialysates as reported by Chen et al.^26,27^. The proteomic profiles were linked with DVT status at two weeks and ATRS at one-year postoperatively for analysis of potential biomarker identification.

### Protein extraction from tissue biopsies and digestions

Tissue samples were pulverized and solubilized in 8 M urea and 100 mM NaCl with 1% ProteaseMAX (Promega) in ammonium bicarbonate (AmBic) and mixed. Low binding silica beads (400 μm, Ops Diagnostics) were added to samples and the samples were therafter vortexed at high speed followed by freezing at -80 °C. After thawing, samples were shakened with a Vortex Genie disruptor for 2 min in two cycles and supplemented with 8 M urea in 50 mM ammonium bicarbonate (AmBic) for protein digestion followed by the addition of 10 µL of 0.2% ProteaseMAX (Promega) in 20% acetonitrile (AcN) and 50 mM AmBic. Additional 220 µL of 50 AmBic was added before determining the protein concentrations by BCA assay (Thermo Scientific). Proteins were reduced with 100 mM dithiothreitol in 50 mM AmBic, incubated at 37 °C and alkylated with 100 mM iodoacetamide in 50 mM AmBic incubated in the dark. Proteolytic digestion was performed with RIPA cell lysis reagent (Thermo Scientific) overnight. The reaction was stopped with concentrated formic acid. The samples were then cleaned on a C18 Hypersep plate (bed volume of 40 µL, Thermo Scientific) and dried in a vacuum concentrator (miVac, Thermo Scientific).

### Protein extraction from microdialysates

The microdialysates were transferred into a 96-well LoBind plate (Applied Biosystems) with 5 µL of 50 mM ammonium bicarbonate (pH = 8.5). Total protein concentration in the microdialysates was assessed with a BCA Protein Assay Kit (Thermo Scientific) according to the manufacturer’s instructions. Protein (5 µg) from each sample was used for subsequent analysis.

### Trypsin digestion and sample alkylation

Trypsin digestion of samples was used to generate peptides of a suitable size for the MS analysis. Ammonium bicarbonate (50 mM) was added up to a total volume of 50 µL. Reduction of disulfide bonds was performed by adding 4 µL of 100 mM dithiothreitol in 50 mM ammonium bicarbonate followed by incubation at 37 degrees for 45 min. Alkylation was performed to prevent the subsequent alkylation of trypsin. Ten µL of 100 mM Iodoacetamide in 50 mM ammonium bicarbonate was added and the samples were incubated for 30 min at room temperature. One point five µL of trypsin (stock concentration 0.2 µg/µl) was added and the samples were incubated overnight at 37 degrees. Acidification was performed by adding 3.5 µL of formic acid (5%). Salts and buffers were removed by using Pierce C18 tips (Thermo Scientific) and Hypersep filter plate (Thermo Scientific). Finally, the samples were dried using a SpeedVac (Thermo Scientific)^26^.

### Proteomic analysis

A 50 cm long C18 EASY-spray and C18 trap columns connected to an Ultimate 3000 UPLC system (Thermo Scientific, USA) were used for the reverse phase liquid chromatographic separation of peptides. The gradient was at set at a flow rate of 300 nL/min and 120 min: 2–26% of buffer B (2% AcN, 0.1% formic acid) for 90 min and up to 95% of buffer B for 5 min. Mass-spectra were acquired on an Q Exactive HF mass spectrometer (Thermo Scientific) in m/z 350 to 1,600 at a resolution of *R* = 120,000 at m/z 200 for full mass, targeting 5 × 10^6^ ions with a maximum injection time of 100 ms, followed by data-dependent higher-energy collisional dissociation (HCD) fragmentations from precursor ions with a charge state 2 + to 7 + of the top 17 most intensive precursors. The tandem mass spectra were acquired with a resolution of *R* = 30,000, targeting 5 × 10^4^ ions with a maximum injection time of 54 ms, setting an isolation width to m/z 1.4 and a normalized collision energy to 28% ^26^.

### Proteomic profile analysis, protein identification and quantification

Raw files were imported to Proteome Discoverer v2.3 (Thermo Scientific) and analyzed using the SwissProt protein database with Mascot v 2.5.1 (MatrixScience Ltd.) search engine^26^. The MS/MS spectra were matched with The Human Uniprot database (last modified: 3 September 2020; ID: UP000005640; 75,777 proteins) using the MSFragger database engine^28^. Searching parameters for MS/MS spectra were (1) up to two missed cleavage sites; (2) peptide mass tolerance of 10 ppm; (3) 0.02 Da for the HCD fragment ions. Carbamidomethylation of cysteine was specified as a fixed modification, whereas oxidation of methionine, deamidation of asparagine and glutamine were defined as variable modifications^26^.

For quantification, both unique and razor peptides were requested. A quality filter was applied (contaminated proteins, low-coverage peptides, failed peptides and peptides that could not be matched with any protein included in the UniProt-database). Only unique peptides ≥ 2% with a false discovery rate (FDR) < 1% were set for identification. The LFQ algorithm implemented was used for protein quantification through MaxQuant software. Perseus v1.6.14.0 was used for identification and preliminary analysis of the proteomic files.

Protein identification was then performed, reverse sequences, contaminants and the “only by site” entries were excluded. Protein abundance was presented by normalized spectrum intensity (LFQ intensity), and an intensity-based absolute quantification (iBAQ) algorithm was used to normalize the data^29,30^. In order to normalize the differences among samples, the iBAQ value of each identified protein was converted to an FOT (fraction of total) value by dividing it by the sum of the LFQ intensity values for all the identified proteins. No-expression markers and empty values among samples were marked with 0 for the LFQ intensity values to validate the data^26^.

After quality filtering, the proteomic profile of biopsies included 855 unique proteins whereof 769 were expressed in patients with poor- and good outcomes, and whereof 738 proteins were expressed in patients with DVT and without DVT. The proteomic profile of microdialysates included 1288 unique proteins after quality filtering, whereof 912 proteins were expressed in patients with poor- and good outcomes, and whereof 738 proteins were expressed in patients with DVT and without DVT^26^.

### Statistical analyses

Patient characteristics were analyzed with a Mann-Whitney U test or Pearson’s chi-squared test, or alternatively a Fisher’s exact test in SPSS (Version 27.0 Armonk, NY: IBM Corp.). To detect differentially abundant proteins in patients with and without DVT at two weeks postsurgically, a fold change (FC) was calculated for all the proteins as a ratio of the mean expression in relation to DVT-status (average expression in DVT group / average expression in non-DVT group) in Excel (Version 16.61.1, Microsoft). Thirty-one biopsy-proteins, and 176 microdialysate-proteins were excluded from analyses because of protein expression only in < 3 patients in a group (Supplementary material; Table S1-2). P-values were calculated in SPSS using the Mann-Whitney U test. A protein was considered downregulated if FC was < 0.5 and *p* < 0.05, and upregulated if FC was > 2 and *p* < 0.05. FC-values were log^2^ transformed and p-values -log^10^ transformed and Volcano plots were created in GraphPad Prism (Version 9.1.2, Dotmatics). Dot plots were created in SPSS. A proportion of zero-values ≥40% was indicated with an asterix (*).

The differentially expessed proteins (DEPs) in relation to DVT were further investigated to evaluate if they also were DEPs in relation to poor patient outcome (ATRS ≤ 80) at one-year postsurgically. Multiple logistic regression analyses were conducted in SPSS, using dichotomized protein expression and patient’s age as independent variables, and DVT or healing outcome as dependent variables. Protein expression levels were dichotomized by median (≤median> ). Area under the curve (AUC) receiver operating characteristic (ROC) was calculated with SPSS to measure performance of the classifiers to predict DVT or no DVT and ATRS≤80 or ATRS > 80. No correction for multiple testing was used as high sensitivity rather than specificity was considered purposeful for this exploratory study.

STRING database version 12.0 (https://string-db.org)^31^ was used for interaction analyses. Gene set enrichment analysis (GSEA) was performed using the GSEA package for R^32^ with GO Biological Process, Molecular Function and Cellular Compartment annotation databases based on log2 fold change. Based on the findings of reduced collagen pathways from GSEA related to both DVT and poor healing, further interaction analyse focused on expanded associations of collagen proteins with DEPs from the biopsy and microdialysate proteomes.

## Results

### Patient characteristics

No significant differences were observed in patient characteristics between those without a DVT (*n = 44*) and those with a DVT (*n = 23*) (Table 1). While calf-IPC is known to reduce the risk of DVT after ATR^1,22,25^, the proportions of different treatments did not significantly influence the distribution of DVT in this study (Table 1). As expected, the ATR cohort was predominantly male (78%), reflecting known sex differences in the ATR incidence^33^. Patients with DVT tended to be slightly older than those without DVT, although the differences were not statistically significant. Patient characteristics for the subgroups assessed (biopsies *n* = 25, microdialysates *n* = 14, both biopsies and microdialysates *n* = 14) are described separately (Supplementary material, Table S3).

**Table 1:** Patient characteristics.

		No-DVT	DVT	*p*-value
Tendon biopsies		*n* = 27	*n* = 12	
Male-sex	n (%)	20 (74)	11 (92)	0.394^§^
Age	M (SD)	40 (8.1)	45 (9.0)	0.091
BMI	M (SD)	26 (3.2)	25 (2.7)	0.988
No nicotine use	n (%)	22 (81)	10 (83)	1.000^§^
TTS (h)	M (SD)	74 (30)	86 (23)	0.270
DS (min)	Median (IQR)	39 (27–49)	37 (32–54)	0.709
Postoperative treatment				0.714
Calf-IPC	n (%)	3 (11)	2 (17)	
VacoPed	n (%)	15 (56)	5 (42)	
Plaster cast	n (%)	9 (33)	5 (42)	
Microdialysates		*n* = 17	*n* = 11	
Male-sex	n (%)	12 (71)	9 (82)	0.668
Age	M (SD)	37 (7.1)	42 (9.7)	0.132
BMI	M (SD)	26 (3.2)	27 (3.4)	0.764
No nicotine use	n (%)	14 (82)	11 (100)	0.258^§^
TTS (h)	M (SD)	78 (33)	71 (30)	0.597
DS (min)	M (SD)	34 (16)	29 (3)	0.363
Postoperative treatment				0.570
Calf-IPC	n (%)	1 (5.9)	2 (18)	
VacoPed	n (%)	8 (47)	5 (45)	
Plaster cast	n (%)	8 (47)	4 (36)	
TSM (days)	M (SD)	15 (2.1)	15 (1.5)	0.544

P-values from Mann-Whitney u-test / Student’s t-test or Fishers exact test^§^. Abbreviations: BMI = Body mass index, TTS = Time from injury to surgery, TSM = Time from surgery to microdialysis, DS = Duration of surgery, DVT = Deep venous thrombosis, IQR = Interquartile range. For TTS tendon biopsies (*n* = 22 no-DVT and *n* = 11 DVT) and microdialysates (*n* = 13 no-DVT and *n* = 8 DVT) and DS tendon biopsies (*n* = 24 no-DVT and *n* = 11 DVT) and microdialysates (*n* = 13 no-DVT and *n* = 8 DVT).

### Proteomic profile of tendon biopsies in relation to DVT

**Fig. 2:**
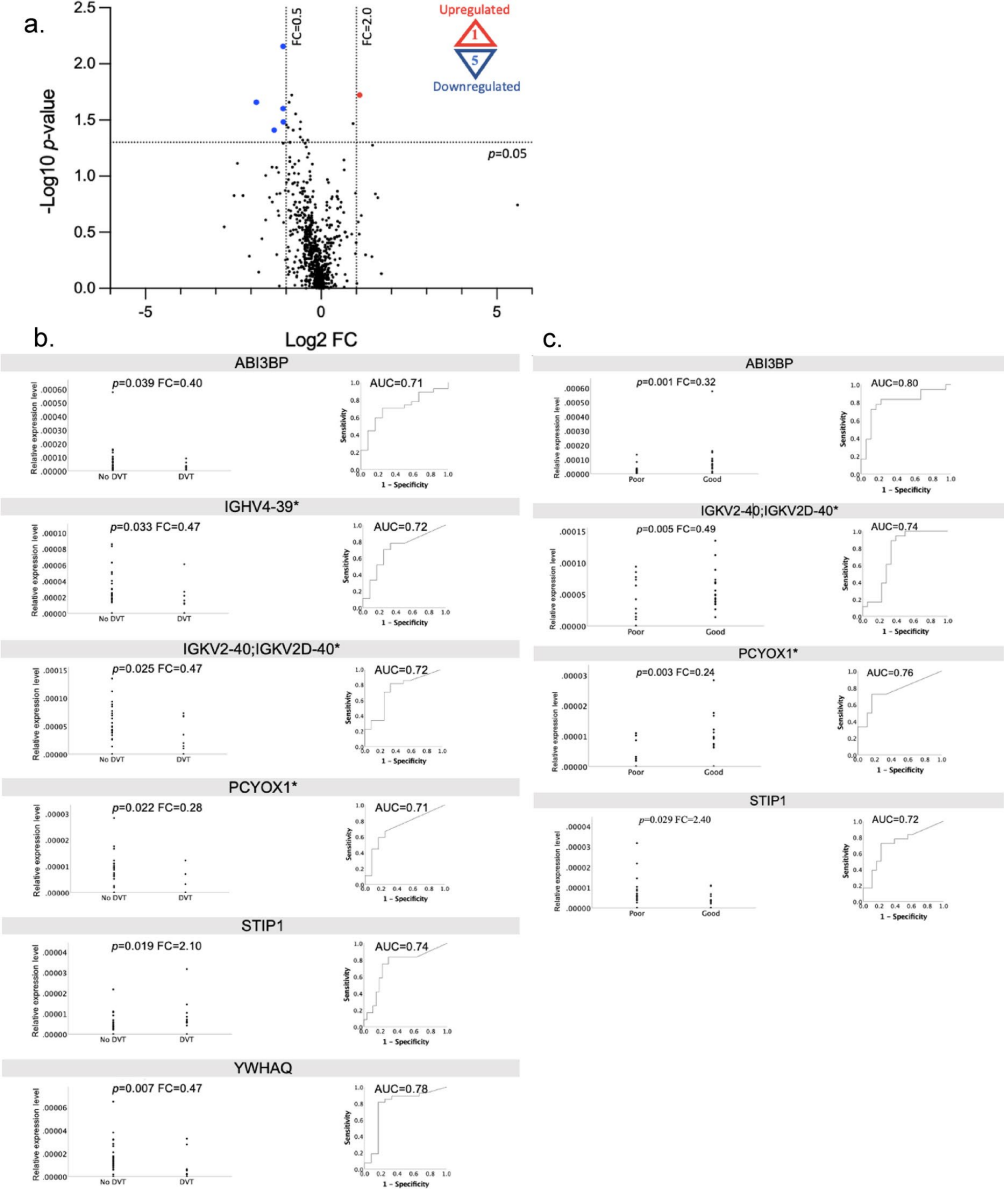
(**a**–**c**) Quantitative proteomics of tendon biopsies reflecting the inflammatory stage of healing. *p*-values from Mann-Whitney U test. *= ≥40% 0 values in good or poor outcome group. Abbreviations: ABI3BP=Abi family member 3 binding protein, AUC= Area under the ROC curve, ROC=Receiver Operating Characteristic, DEP = differentially expressed proteins, FC=Fold-change, IGHV4-39= Immunoglobulin heavy variable 4-39, IGKV2-40/IGKV2D-40= Immunoglobulin kappa variable 2-40/2D-40, PCYOX1= Prenylcysteine oxidase 1, STIP1= Stress-induced-phosphoprotein 1, YWHAQ= Tyroseine 3/Tryptophan 5 Monoxygenase activation protein theta. (**a**). Volcano plot of tendon biopsy proteins. DEPs in patients with DVT (*n* = 12) compared to patients without DVT (*n* = 27) at two weeks after surgery. The *X* coordinate represents Log2 (FC) and the *Y* coordinate represents −Log10 (*p*-value). Each dot represents a protein with red = up-regulated (*n* = 1; STIP1), blue = down-regulated (*n* = 5; ABI3BP, IGHV4-39*, IGKV2-40/IGKV2D-40*, PCYOX1*, and YWHAQ) and, black = non-differentially expressed proteins, (**b**). Dot-plots and ROC curves for DEPs in relation to DVT at two weeks after surgery. For STIP1, ROC when predicting DVT while for other proteins when predicting no DVT, (**c**). Dot-plots and ROC curves for DEPs in relation to DVT at one-year after surgery. For STIP1, ROC when predicting poor outcome (ATRS≤80) while for other proteins when predicting good outcome (ATRS>80).

Analysis of 738 proteins from tendon biopsies identified six DEPs among 27 patients without DVT and 12 patients with DVT (Fig. 2). One protein, Stress-induced-phosphoprotein 1 (STIP1) was significantly upregulated, while five proteins - ABI3BP, IGHV4-39, IGKV2-40/IGKV2D-40, PCYOX1, and YWHAQ - were significantly downregulated in patients with DVT two weeks postoperatively (Fig. 2).

In the logistic regression analyses adjusted for age, five of the six DEPs were significantly associated with the DVT status at two weeks postoperatively (Table 2). Higher levels of STIP1 increased the odds of a DVT (OR = 8.40, *p* = 0.019), while higher levels of ABI3BP, IGHV4-39, IGKV2-40/IGKV2D-40, and YWHAQ decreased the odds of DVT (Table 2). All six proteins demonstrated predictive value for DVT, with area under the curve (AUC) values exceeding 0.70 (Fig. 2).

### Shared biomarker candidates for DVT and healing from tendon biopsies

**Fig. 3:**
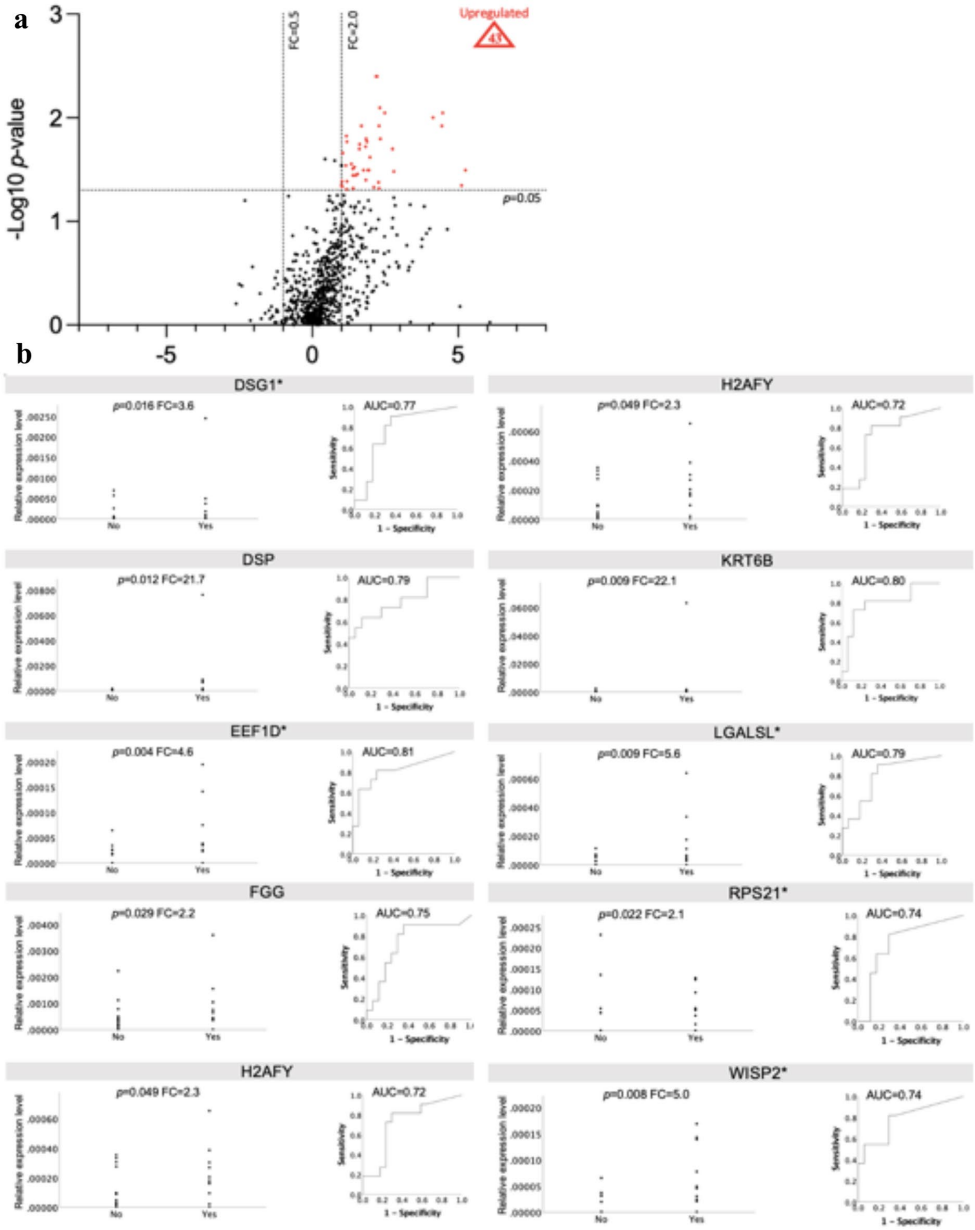
(**a**–**b**). Quantitative proteomics of tendon microdialysates reflecting the proliferative stage of healing. *p*-values from Mann-Whitney U test. The proportion of 0-values ≥40% in any group indicated with an asterix (*). Abbreviations: DEP = differentially expressed protein, DSG1= Desmoglein-1, DSP= Desmoplakin, DVT=Deep venous thrombosis, EEF1D= Elongation factor 1-delta, EEF2= Elongation factor 2, FC=Fold-change, FGG=Fibrinogen gamma chain, H2AFY= Core histone macro-H2A.1, KRT6B= Keratin, type II cytoskeletal 6B, LGALSL=Galectin related protein, ROC=Receiver Operating Characteristic, RPS21=40 S ribosomal protein S21, WISP2= CNN family member 5. (**a**). Volcano plot of tendon biopsy proteins. DEPs in patients with DVT (*n* = 11) compared to patients without DVT (*n* = 17) at two weeks post-surgery. The *X* coordinate represent Log2 (FC) and the *Y* coordinate to −Log10 (*p*-value). Each dot represents a protein with red = up-regulated (*n* = 43) and, black = non-differentially expressed proteins. List of all microdialysate DEPs in supplementary materials S6. (**b**). Dot-plots and ROC curves for the ten most significant DEPs from microdialysates in relation to DVT at two weeks after surgery.

Further analysis revealed that four of the six DEPs associated with DVT were also differentially expressed in relation to healing outcome at one-year postoperatively. STIP1 was significantly upregulated, while ABI3BP, IGKV2-40/IGKV2D, and PCYOX1 were significantly downregulated in patients who exhibited a poor outcome (ATRS≤80) (Fig. 3).

In logistic regression analyses adjusted for age, elevated STIP1 levels significantly increased the odds of a poor outcome, while elevations in ABI3BP, IGKV2-40/IGKV2D-40, and PCYOX1 reduced the odds of a poor outcomes (Table 3, the unadjusted values and proportion of 0-values in supplementary materials S4 and S5). All of the four proteins exhibited AUC values > 0.70 in prediction of healing outcomes (Fig. 3).

**Table 2:** DEPs from tendon biopsies prognostic for DVT at 2 weeks and poor healing outcome at one-year post-surgery.

	DVT at 2 weeks		Poor healing outcome at 1 year	
	OR (95% CI)	*p*	OR (95% CI)	*p*
ABI3BP	0.19 (0.04–0.95)	**0.042**	0.06 (0.01–0.30)	**0.001**
IGHV4-39*	0.16 (0.03–0.82)	**0.028**		
IGKV2-40/IGKV2D-40*	0.14 (0.02–0.77)	**0.024**	0.24 (0.06–0.98)	**0.047**
PCYOX1*	0.21 (0.04–1.03)	0.054	0.11 (0.02–0.4)	**0.004**
STIP1	8.40 (1.40–50.0)	**0.019**	5.09 (1.20–21.0)	**0.026**
YWHAQ	0.09 (0.01–0.59)	**0.012**		

DVT at 2 weeks (*n* = 39) and poor healing at 1 year (*n* = 36). Logistic regression adjusted for patient age. Protein expression dichotomized by median. The proportion of 0-values ≥40% in any group indicated with an asterix (*). Abbreviations: ATRS = Achilles tendon Total Rupture Score, DEP = differentially expressed proteins, DVT = Deep Venous Thrombosis, OR = Odds ratio, 95% CI = 95% confidence intervall for the odds ratios. ABI3BP = Abi family member 3 binding protein, IGHV4-39 = Immunoglobulin heavy variable 4–39, IGKV2-40/IGKV2D-40 = Immunoglobulin kappa variable 2–40/2D-40, PCYOX1 = Prenylcysteine oxidase 1, STIP1 = Stress-induced-phosphoprotein 1, YWHAQ = Tyroseine 3/Tryptophan 5 Monoxygenase activation protein theta.

### Proteomic profiling of tendon microdialysates in relation to DVT and healing outcomes

Analysis of 738 proteins in tendon microdialysates identified 43 DEPs among 17 patients without a DVT and 11 patients with a DVT, all of which were upregulated in patients with a DVT (Fig. 3a).

In logistic regression analyses adjusted for age, 10 of these DEPs (DSG1, DSP, EEF1D, EEF2, FGG, H2AFY, KRT6B, LGALSL, RPS21, and WISP2) significantly increased the odds of having a DVT (Table 3, the unadjusted values in supplementary materials S7). All 10 proteins exhibited AUC values > 0.70 in prediction of DVT (Fig. 3b). Further analysis revealed no relation of these 43 DEPs with healing outcome at one-year post-surgery.

**Table 3:** The ten significant DEPs from microdiaysates prognostic for DVT at 2 weeks post-surgery.

	DVT at 2 weeks	
	OR (95% CI)	*p*
DSG1*	9.5 (1.4–63.0)	**0.020**
DSP	8.4 (1.1–64.0*)*	**0.040**
EEF1D*	9.2 (1.4–61.0)	**0.021**
EEF2*	9.0 (1.3–63.0)	**0.027**
FGG	12.0 (1.7–86.0)	**0.014**
H2AFY	11.0 (1.6–78.0)	**0.014**
KRT6B	15.0 (1.8–130.0)	**0.013**
LGALSL*	19.0 (2.0-180.0)	**0.010**
RPS21*	9.0 (1.3–61.0)	**0.026**
WISP2*	9.0 (1.3–63.0)	**0.027**

DVT at 2 weeks (*n* = 39). Logistic regression adjusted for age. Protein expression dichotomized by median. The proportion of 0-values ≥40% in proteins indicated with an asterix (*) Abbreviations: DEP = differentially expressed proteins, DVT = Deep Venous Thrombosis, OR = Odds ratio, 95% CI = 95% confidence intervall for the odds ratio, DSG1 = Desmoglein-1, DSP = Desmoplakin, EEF1D = Elongation factor 1-delta, EEF2 = Eukaryotic elongation factor 2, FGG = Fibrinogen gamma chain, H2AFY = Core histone macro-H2A, KRT6B = Keratin, type II cytoskeletal 6B, LGALSL = Galectin-related protein, RPS21 = Ribosomal protein S21, WISP2 = WNT1-inducible-signaling pathway protein 2.

### Protein-protein interactions among DEPs from biopsies and microdialysates

**Fig. 4:**
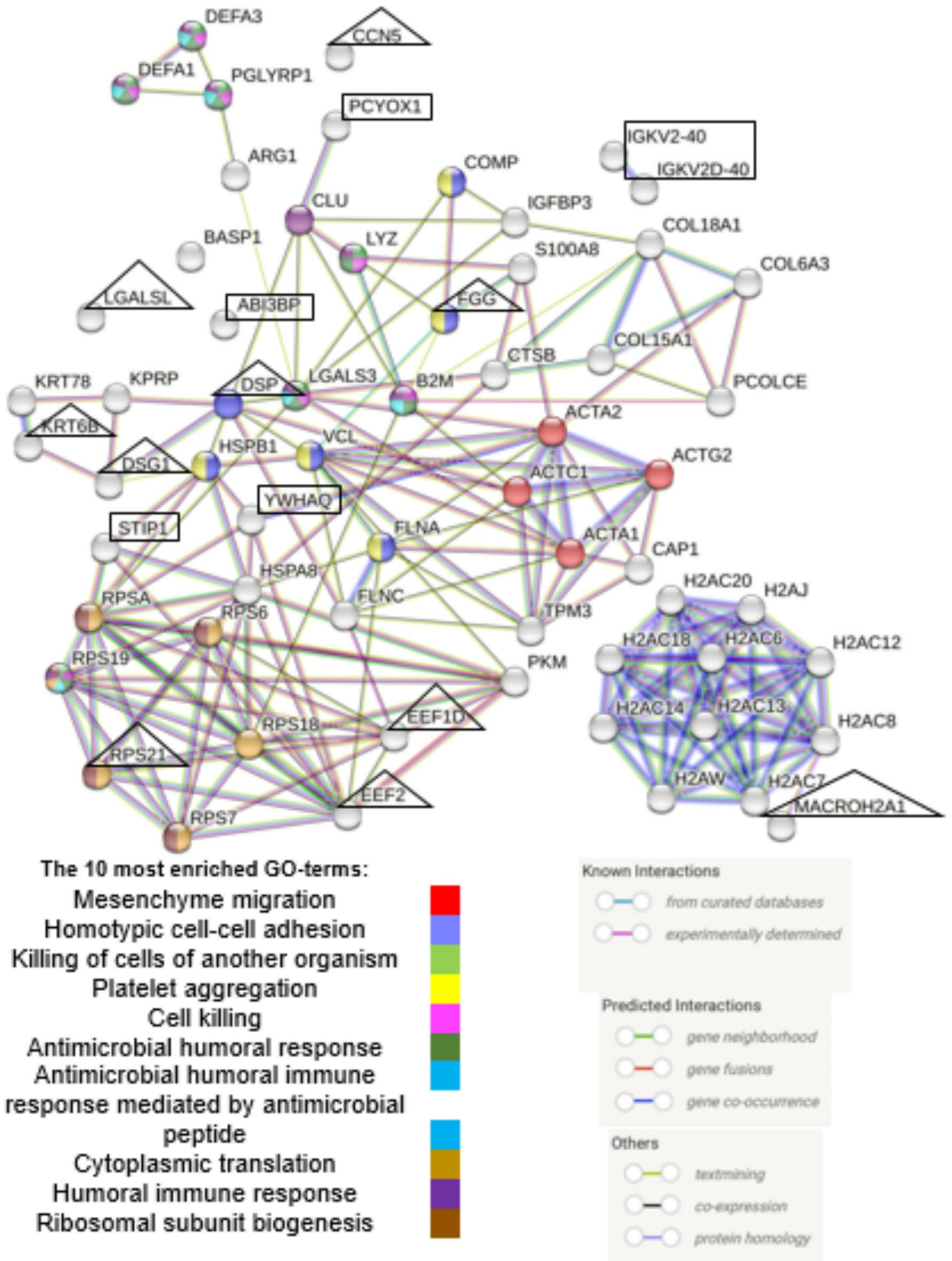
Protein-protein interactions among differentially expressed tissue- and microdialysate proteins. Line thickness = strength of evidence. Marked with a rectangle = biomarker candidates from tendon biopsies (IGHV4-39 unavailabale). Marked with triangle = the ten most robust biomarker candidates from tendon microdialysates. Abbreviations: ACTA1*=Actins (ACTA1/ACTC1/ACTA2/ACTG2), ARG1*=Arginase 1, B2M=Beta-2-mikroglobulin, BASP1*=Brain acid soluble protein 1, CAP1=Adenylyl cyclase-associated protein 1, CLU=Clusterin, COL15A1*= Collagen, type XV, alpha 1, COL18A1=Collagen, type XVIII, alpha 1, COL6A3=Collagen, type VI, alpha 3, COMP=Cartilage oligomeric matrix protein, CTSB=Cathepsin B, DEFA3/DEFA1*= Defensin, alpha 3 / alpha1, DSG1*=Desmoglein-1, DSP=Desmoplakin, EEF1D*=Elongation factor 1-delta, EEF2*=Eukaryotic elongation factor 2, FGG=Fibrinogen gamma chain, FLNA=Filamin A, FLNC*=Filamin C, GO=Gene Ontology, H2AFY=MACROH2A1=Core histone macro-H2A, H2AC14*=Histones (HIST1H2AJ/HIST1H2AH/H2AFJ/HIST2H2AC/HIST1H2AC/HIST3H2A/ HIST2H2AA3/HIST1H2AD/HIST1H2AG/HIST1H2AB), HSPA8=Heat shock protein family A member 8, HSPB1*=Heat shock protein family B member 1/ heat shock protein 27, IGFBP3*=Insulin-like growth factor-binding protein 3, KPRP*=Keratinocyte proline rich protein, KRT6B=Keratin, type II cytoskeletal 6B, KRT78*=Keratin, type II cytoskeletal 78, LGALS3*=Galectin-3, LGALSL*=Galectin-related protein, LYZ=Lysozyme, PCOLCE=Procollagen C-endopeptidase enhancer, PGLYRP1*=Peptidoglycan recognition protein 1, PKM=Pyruvate kinase, RPS6*=Ribosomal protein S6, RPS7*=Ribosomal protein S7, RPSA*=Ribosomal protein SA, RPS18*=Ribosomal protein S18, RPS19*= Ribosomal protein S19, RPS21*=Ribosomal protein S21, S100A8*=S100 calcium-binding protein A8, TPM3*=Tropomyosin 3, VCL*=Vinculin, WISP2*=CCN5=WNT1-inducible-signaling pathway protein 2.

No direct interactions were identified among the six DVT-associated DEPs from tissue biopsies (Fig. 4). Interaction data was unavailable for one protein (IGHV4-39)^31^. In contrast, multiple interactions were found among the 43 DVT-associated DEPs from microdialysates (Fig. 4). Integrative analysis revealed several interactions between biopsy-derived DEPs (e.g. STIP1 and YWHAQ) and microdialysate-derived DEPs (e.g. HSPA8, HSBP1, and EEF2), all of which are linked to the heat shock response and may contribute to collagen metabolism^34^. PCYOX1 from biopsies exhibited a direct interaction with CLU from microdialysates, a cellular chaperone with similar functions as heat shock proteins^35^. ABI3BP remained isolated in the interaction map (Fig. 4).

### Gene set enrichment analysis (GSEA)

**Fig. 5:**
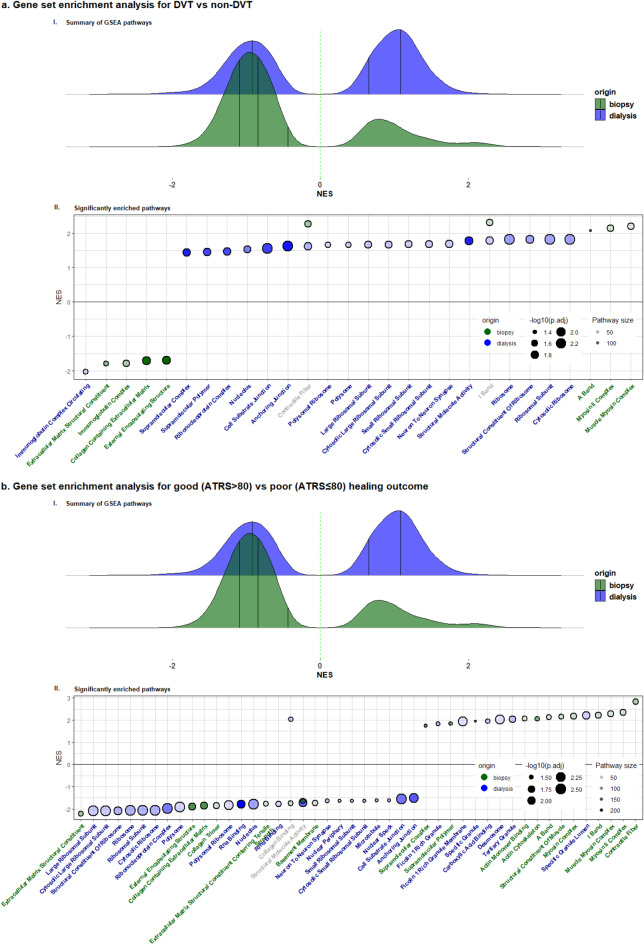
(**a**,**b**). GSEA for DVT and healing outcome measured with ATRS. (I) A ridge plot of pathway enrichment on the x-axis separated by origin (biopsy or microdialysis). (II) The top 20 pathways (in both directions) from biopsies (green) and microdialysates (blue) – Pathway enrichment on the y axis, pathway names on the x-axis. Color is origin, dot size is the p-value, and transparency is the size of the pathway. Abbreviations: GSEA = Gene set enrichment analysis, NES = Normalized enrichment score.

In tendon biopsies, DVT and poor healing outcomes were associated with fewer enriched pathways compared to non-DVT and favorable healing outcomes, while the opposite was observed in microdialysates (Fig. 5a-b). In biopsies, pathways related to collagen synthesis, extracellular matrix (ECM), and immunoglobulin complex were reduced in the DVT group (Fig. 5a). In microdialysates, only the immunoglobulin complex pathway was reduced in association with the development of DVT, while most enriched pathways were related to ribosomal proteins (Fig. 5a). For poor healing outcomes, biopsies revealed reductions in collagen synthesis and extracellular matrix pathways while the most enriched pathways were mainly associated with structural and developmental muscle terms (Fig. 5b) demonstrating remarkable similarity with the DVT-enriched pathways. In microdialysates, the most reduced pathways in relation to poor healing outcome were mainly ribosome related but also collagen related, while the most enriched pathways were less specific (Fig. 5b).

#### Extended collagen pathways with DEPs from biopsy and microdialysate proteomes

**Fig. 6:**
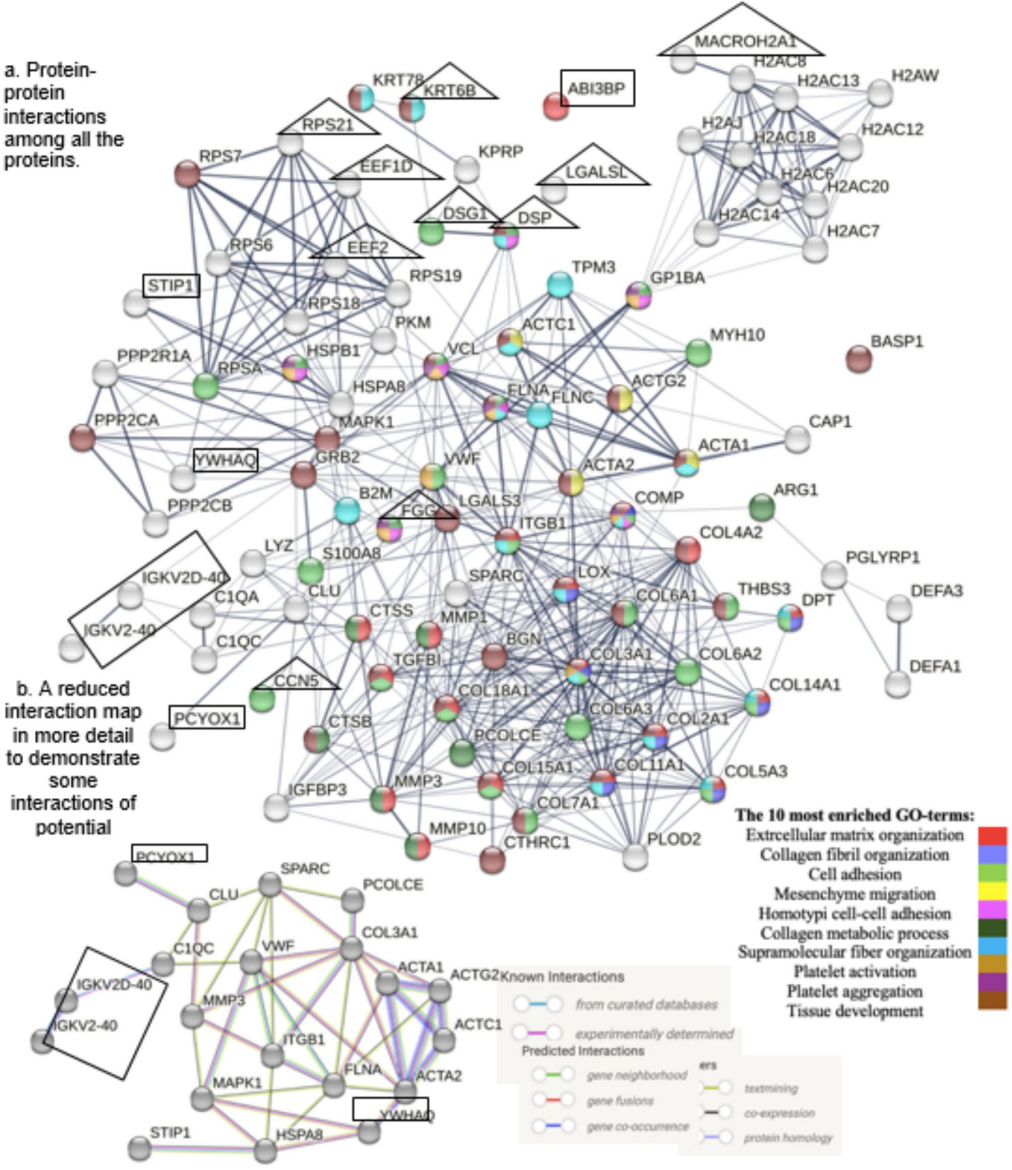
(**a**,**b**). Protein-protein interactions among DVT associated DEPs from biopsies and microdialysates, and collagen proteins from biopsies and microdialysates. DEPs = Differentially expressed proteins. Marked with a rectangle = biomarker candidates from tendon biopsies (IGHV4-39 unavailable). Marked with triangle = the ten most robust biomarker candidates from tendon microdialysates. H2AFY=MACROH2A1, WISP2=CCN5.

Proteomic analysis identified 22 collagen-related proteins in biopsies and 14 in microdialysates, expanding the the ECM related network of collagen synthesis and modification and revealed further pathways involved in DVT and healing (Fig. 6)^31^. PCYOX1 (biopsies), interacted via CLU (microdialysates) with Osteonectin (SPARC), which regulates cell growth through interactions with the ECM and cytokines. SPARC interacted with Von Willebrand Factor (VWF), a molecule that promotes adhesion of platelets to the sites of injury in hemostasis (Fig. 6). YWHAQ (biopsies), interacted with VWF via Mitogen-Activated Protein Kinase 1 (MAPK1), an essential component in the MAPK/ERK cascade and mediator of diverse biological functions such as cell growth, adhesion, survival and differentiation (Fig. 6).

COL3A1, i.e. collagen type III, is the dominating collagen type during the early proliferative stage of tendon healing^10,36^. The interaction map demonstrated several DEPs from biopsies, e.g. STIP1 and YWHAQ, interacting with COL3A1, via e.g. HSPA8, ITGB1, and VWF (Fig. 6b). Of the DEPs from microdialysates, PCOLCE, ACTA, and FLNA interacted directly with COL3A1 (Fig. 6b).

## Discussion

Proteomic profiles reflecting the inflammatory- and proliferative stages of Achilles tendon rupture (ATR) healing, i.e., the intraoperative tendon biopsies and microdialysates collected at two weeks postoperatively, along with their interactions, were linked with deep venous thrombosis (DVT) and clinical patient outcome data. Six candidate DVT biomarkers were detected from biopsies, whereof four also were candidate biomarkers for healing outcome. These findings add to existing knowledge on the thrombotic processes and parallel healing pathways during lower-limb immobilization and suggest new therapeutic targets to prevent both DVT and healing disturbances.

An important discovery of the present study was the identification of six novel candidate DVT biomarkers from biopsies: ABI3BP, IGHV4-39, IGKV2-40/IGKV2D-40, PCYOX1, STIP1, and YWHAQ. Literature supports the involvement of all the six proteins in thrombotic processes^36–44^, and for several of these proteins, also in redox signaling. The present findings indicated that all except PCYOX1, affected the odds of developing a DVT independent of age, and predicted onset of a DVT with an acceptable precision^45^, strengthened the role of these proteins in venous thrombosis development.

Another essential finding was the dual role of four biopsy-derived DVT-biomarkers (ABI3BP, IGKV2-40/IGKV2D-40, PCYOX1, and STIP1) in predicting poor healing outcomes, and were independent of age^45^. The observation that increases in STIP1, and decreases in the other three proteins, predicted the development of a DVT as well as a poor healing outcome, were congruent with the hypothesis posed in the present study and based on previous evidence that developing a DVT is associated with inferior patient outcomes after ATR^6–8^.

Forty-three candidate DVT-biomarkers were identified in microdialysates, eight of which were previously detected from a fibrin clot proteome of patients with pulmonary embolism^46^, and one from the plasma of trauma-immobilized patients with venous thrombosis^47^. Our findings demonstrated that ten of the 43 candidate DVT-biomarkers from microdialysates significantly increased the odds of a DVT independent of age, and exhibited acceptable predictive capacity for a DVT^45^. Six of the ten proteins have previously reported roles in thrombotic processes^46,48,49^.

Protein-protein interaction analyses together with gene set enrichment analysis (GSEA) emphasized roles for underactive antigen recognition, extracellular matrix (ECM) and collagen downregulation in DVT and healing disturbances. Downregulation of genes that encode ECM in DVT were recently reported in a rat model^50^ and downregulation of collagen-metabolism in healing disturbances is consistent with the current knowledge^25,51^. Interaction analyses further highlighted downstream signaling via heat shock proteins (HSPs) and nitric oxide (NO), not only in thrombotic processes but in tissue healing as well, presumaby generating variations in abilities to counteract and tolerate hypoxic conditions e.g., during lower-leg immobilization after ATR^15–18,44,52–59^.

Several biomarkers, including PCYOX1 and YWHAQ, were previously implicated in platelet signaling^37,41^ which presumably is a central concept in thrombosis and healing^52^. STIP1, a candidate biomarker for DVT and healing outcome from biopsies, serves as a cofactor for the molecular chaperones HSP70 and HSP90 ^38^. HSP70 has been reported to modify platelet signaling^39,40^ and HSP90 to upregulate nitric oxide (NO)^15,53^ with platelet-regulatory, vasodilatatory, and healing promoting properties^54,55^. It has been suggested that STIP1-independent interaction of HSP70 and HSP90 may result in superior chaperoning activity^38^ which aligns with the present observations linking STIP1 to DVT and poor healing outcome. Alternatively, STIP1 may vary in response to local hypoxia, modulated by lower-limb immobilization and individual tolerance. The relevance of tissue hypoxia in ATR patients is strenghtened by earlier findings associating tendon microcirculation during immobilization with patient outcome^56,57^.

The main study limitations include methodological differences in the study of biopsy- and microdialysis-based proteomics, a high proportion of zero-values, lack of baseline DVT diagnostics, and variability in post-operative rehabilitation regimens. Additionally, the cohort size limited stratifying analysis by sex. However, these study limitations must be considered in the light of its explorative nature, being the first human study using proteomics of ATR-tissue to predict DVT development and long-term healing outcomes. Nevertheless, this study had several advantages, such as the integrated use of liquid chromatography-tandem mass spectrometry, ultrasonography for DVT screening, and longitudinal sampling of materials from two time points after tendon injury.

## Conclusions

Several candidate prognostic biomarkers for deep venous thrombosis during early lower leg immobilization, as well as for reported healing disturbances after Achilles tendon rupture were identified from a proteomic landscape of human dense connective tissue. These findings are novel and support shared protein pathways in the pathophysiology of venous thrombosis and poor tendon healing reported outcomes. Key roles for individual hypoxic tolerance and platelet signaling were suggested, providing potential targets for future therapeutic interventions. Further research is warranted to confirm these findings and to target the molecular pathways that could lead to new clinical therapies to mitigate these adverse risks.

## Electronic supplementary material

Below is the link to the electronic supplementary material.

Supplementary Material 1

Supplementary Material 2

Supplementary Material 3

## Data Availability

The data that support the findings of this study are available in Supplementary Material (Table S8-9).

## Abbreviations

ABI3BP: Abi family member 3 binding protein
ATR: Achilles tendon rupture
ATRS: Achilles tendon total rupture score
AUROC: Area under receiver operating characteristic curve
DEP: Differentially expressed proteins
DVT: Deep venous thrombosis
GSEA: Gene Set Enrichment Analysis
HSR: Heat Shock response
HSPS: Heat shock proteins
IGHV4-39: Immunoglobulin heavy variable 4–39
IGKV2-40; IGKV2D-40: Immunoglobulin kappa variable 2–40 and 2D-40
IPC: Intermittent pneumatic compression
MS: Heat Shock response
PCYOX1: Prenylcysteine oxidase 1
ROC: Receiver operating characteristic
STIP1: stress-induced-phosphoprotein 1.
YWHAQ: yrosine 3-monooxygenase/tryptophan 5-monooxygenase activation protein theta
