# Supplementary material for: Novel tissue biomarker candidates to predict both deep venous thrombosis and healing outcome after Achilles tendon rupture
Source: Sci Rep. 2025 Mar 1;15:7318. doi: 10.1038/s41598-025-91511-0 (PMC11873306; doi:10.1038/s41598-025-91511-0)
Supplement: Supplementary file 1 — Supplementary Material 1 [file 41598_2025_91511_MOESM1_ESM.docx]

**Table S1.** The biopsy proteins excluded from analyses because of expression in ≤3 patients in a group (DVT and/or no-DVT).

| ACAT1  ACTN2  ACTN3  ADH1A  ADH1B  ADH1C  ALDOC  ANPEP | CAPZB  FNDC1  GLUL  GOT2  GSTT1  HMGB1  IGHV3-23  IGKV1D-8 | INA  KANK2  LAMB2  LMAN1  MMP2  MYH11  NGEF  PLIN4 | RAB18  RETN  RPN1  RPS19  SSB  TNXA  TSTA3 |
| --- | --- | --- | --- |

**Table S2.** The microdialysate proteins excluded from analyses because of expression in only ≤3 patients in a group (DVT and/or no-DVT).

| ABCF1  ACTN4  AHSP  AKAP12  ALAD  ANGPTL2  ANP32A  ANXA3  AP2B1  AP3D1  APOF  APOM  ARFGAP3  ARL3  ARPP19  ATXN2L  BAX  BCHE  BCL2L13  BIN1  BMP1  BPIFA1  C1QA  C1RL  C20orf27  CAB39  CALB2  CAMP  CANX  CAV1  CD74 CFHR3/CFHR4  CHAD  CHIT1  CIAPIN1  CILP2  CKB | CKM  COL4A2  COX6B1  CRYAB  CSRP3  CYB5A  DAB2  DDAH1  DDX39B  DES  DMTN  DNAJC1  DNPH1  DYNC1LI1  EDF1  EIF2A  EIF3J  GART  GOT1  GP1BA  GSTM3  GSTT1  H1FX  HAL  HBG2  HBQ1  HBZ  HMGA1  HNMT  HNRNPH3  HNRNPR  HSP90AB1  HTRA1  IGHV4-61/IGHV4-4/IGHV4-31  IGHV5-51 | IGKV A18/IGKV2D-26/IGKV2D-29/IGKV2D-30/IGKV2D-28/IGKV2-40  IGKV3D-15/IGKV3OR2-268/IGKV3-7/IGKV3D-7  IGHV3-23  IGLV3-10  IMPDH2  ISG15  ITIH3  KRT13  LBP  LCN1/LCN1P1  LDB3  LILRB5/LILRB2  LOX  LUC7L2  MAP1A  MAPK1  MASP2  MB  MBNL1  MMP3  MUSTN1  MYBPC1  MYH7;MYH6  MYLK  MYOF  MYOM2  NAGA  NEB  NEXN | NONO  NOP58  NR2C2AP  NUDT2  OGN  OLA1  OLFM4  P4HB  PA2G4  PALM  PDLIM2  PEBP4  PGM2  PHYHD1  PKP1  PLAUR  PML  POF1B  PRG4  PROSC  PRR4  PRTN3  PSMA6  RAB27B  RBMXL1  RNASET2  RPL27  RPL8  RPLP2  RPS24  RPS27  RRAS  RSL1D1  RTCA  S100A1  S100A9  SCAMP3 | SEC31A  SERPINA7  SERPINB12  SF1  SFPQ  SH3PXD2B  SMNDC1  SMU1  SNRNP200  SNRPA  SPRR1B  SRGN  SRRM1  STIP1  TARDBP  TGM3  TMEM263  TMX1  TNNC1  TNNT1  TPMT  TRAM1  TUBA4A  TWF  TYMP  UBA1  UBE2I  UBE2V2  UROS  USP15  VPS29  WDR44  WIBG  WIPF1  XRCC6  YKT6  ZC3HAV1  ZG16B |
| --- | --- | --- | --- | --- |

**Table S3.** Patient characteristics described separately in patients with only tendon biopsies (n=25), only microdialysates (n=14) or tendon biopsies as well as microdialysates (n=14).

|  | | No-DVT | DVT | p-value |
| --- | --- | --- | --- | --- |
| **Tendon biopsies** | | n=17 | n=8 |  |
| Male-sex | n (%) | 13 (77) | 7 (89) | 1.000* |
| Age | Median (IQR) | 38 (9) | 48 (16) | 0.085 |
| BMI | M (SD) | 25 (4) | 26 (5) | 0.641 |
| Nicotine use | n (%) | 3 (18) | 2 (25) | 1.000* |
| TTS (h) | Median (IQR) | 63:06 (48:37) | 86:14 (25:49) | 0.133 |
| DS (min) | Median (IQR) | 0:39 (0:19) | 0:42 (0:20) | 0.426 |
| Calf-IPC | n (%) | 3 (18) | 2 (25) | 1.000* |
| VacoPed | n (%) | 8 (47) | 2 (25) | 0.402* |
| Plaster cast | n (%) | 6 (35) | 4 (50) | 0.667* |
| **Microdialysates** | | n=7 | n=7 |  |
| Male-sex | n (%) | 5 (71) | 5 (71) | 1.000* |
| Age | Median (IQR) | 36 (6) | 47 (19) | 0.179 |
| BMI | Median (IQR) | 25 (3) | 25 (8) | 0.749 |
| Nicotine use | n (%) | 1 (14) | 0 (0) | 1.000* |
| TSM (d) | Median (IQR) | 17 (6) | 17 (-) | 0.759 |
| TTS (h) | Median (IQR) | 63:42 (39:08) | 67:05 (59:02) | 0.754 |
| DS (min) | Median (IQR) | 0:27 (0:31) | 0:30 (0:04) | 0.599 |
| Calf-IPC | n (%) | 1 (14) | 2 (29) | 1.000* |
| VacoPed | n (%) | 1 (14) | 2 (29) | 1.000* |
| Plaster cast | n (%) | 5 (71) | 3 (43) | 0.592 |
| **Tendon biopsies and microdialysates** | | n=10 | n=4 |  |
| Male-sex | n (%) | 7 (70) | 4 (100) | 0.505 |
| Age | Median (IQR) | 39 (8) | 40 (19) | 0.777 |
| BMI | Median (IQR) | 25 (4) | 25 (4) | 0.944 |
| Nicotine use | n (%) | 2 (20) | 0 (0) | 1.000* |
| TTM (d) | Median (IQR) | 14 (1) | 16 (-) | 0.053 |
| TTS (h) | Median (IQR) | 90:34 (43:18) | 88:44 (-) | 0.838 |
| DS (min) | Median (IQR) | 0:36 (0:22) | 0:29 (-) | 0.838 |
| Calf-IPC | n (%) | 0 (0) | 0 (0) | - |
| VacoPed | n (%) | 7 (70) | 3 (75) | 1.000* |
| Plaster cast | n (%) | 3 (30) | 1 (25) | 1.000* |

Some of the patients did both tendon biopsies and microdialysis while some did only either tendon biopsies or microdialysis. BMI=Body mass index TTS= Time from injury to surgery DS=Duration of surgery TTM=Time from surgery to microdialysis IQR=Interquartile range. P-values from Mann-Whitney test or Chi-squared test / Fisher’s exact test*. TTS biopsies (n=14 no-DVT), DS biopsies (n=16 no-DVT), TSM microdialysates (n=5 no-DVT and n=3 DVT), TTS microdialysates (n=5 no-DVT and n=5 DVT), DS microdialysates (n=5 no-DVT and n=5 DVT), TTM biopsies and microdialysates (n=8 no-DVT and n=3 DVT), TTS tendon biopsies and microdialysates (n=8 no-DVT and n=3 DVT), and DS tendon biopsies and microdialysates (n=8 no-DVT and n=3 DVT).

| **Table S4.** Table of unadjusted odds ratios, 95% confidence intervals and p-values for DVT at 2 weeks and poor outcome at 1 year. | | | | | |
| --- | --- | --- | --- | --- | --- |
|  |  | DVT at 2 weeks^n=39^ | | Poor outcome at 1 year^n=36^ | |
|  |  | OR (95% CI) | *p* | OR (95% CI) | *p* |
| ABI3BP |  | 0.20 (0.04-0.90) | ***0.036*** | 0.06 (0.01-0.30) | ***0.001*** |
| IGHV4-39* |  | 0.20 *(0.04-0.90)* | ***0.036*** |  |  |
| IGKV2-40/IGKV2D-40* |  | 0.20 *(0.04-0.90)* | ***0.036*** | 0.25 (0.06-1.00) | *0.050* |
| PCYOX1* |  | 0.23 (0.05-1.04) | *0.057* | 0.11 (0.02-0.50) | ***0.004*** |
| STIP1 |  | 8.50 (1.50-47.0) | ***0.014*** | 5.20 (1.30-22.0) | ***0.023*** |
| YWHAQ |  | 0.12 (0.02-0.65) | ***0.014*** |  |  |
| Logistic regression adjusted for age. Protein expression dichotomized by median. The proportion of 0-values ≥40% in proteins indicated with an asterix (*) Abbreviations: ATRS=Achilles tendon Total Rupture Score, DEP = differentially expressed proteins, DVT= Deep Venous Thrombosis, DEP = differentially expressed proteins, OR= Odds ratio, 95% CI = 95% confidence interval for the odds ratio. ABI3BP=Abi family member 3 binding protein, IGHV4-39= Immunoglobulin heavy variable 4-39, IGKV2-40/IGKV2D-40= Immunoglobulin kappa variable 2-40/2D-40, PCYOX1= Prenylcysteine oxidase 1, STIP1= Stress-induced-phosphoprotein 1, YWHAQ= Tyrosine 3/Tryptophan 5 Monooxygenase activation protein theta | | | | | |

| **Table S5.** Proportion of 0-values for tendon biopsy-derived DEPs. | | | |
| --- | --- | --- | --- |
| % | Total | No-DVT | DVT |
| ABI3BP | 0 | 0 | 0 |
| IGHV4-39 | 28 | 19 | 50 |
| IGKV2-40/IGKV2D-40 | 23 | 15 | 42 |
| PCYOX1 | 45 | 33 | 75 |
| STIP1 | 28 | 33 | 17 |
| YWHAQ | 13 | 7 | 25 |

DEP=Differentially expressed protein

| **Table S6.** List of up-regulated microdialysate proteins of Figure 3. | |
| --- | --- |
| ACTA1/ACTC1/ACTA2/ACTG2* = Actins | HSPB1* = Heat shock protein family B member 1 |
| ARG1* = Arginase 1 | IGFBP3* = Insulin-like growth factor-binding protein 3 |
| B2M = Beta-2-mikroglobulin | KPRP* = Keratinocyte proline rich protein |
| BASP1* = Brain acid soluble protein 1 | KRT6B = Keratin, type II cytoskeletal 6B |
| CAP1 = Adenylyl cyclase-associated protein 1 | KRT78* = Keratin, type II cytoskeletal 78 |
| CLU = Clusterin | LGALS3* = Galectin-3 |
| COL15A1* = Collagen, type XV, alpha 1 | LGALSL* = Galectin-related protein |
| COL18A1 = Collagen, type XVIII, alpha 1 | LYZ = Lysozyme |
| COL6A3 = Collagen, type VI, alpha 3 | PCOLCE = Procollagen C-endopeptidase enhancer |
| COMP = Cartilage oligomeric matrix protein | PGLYRP1* = Peptidoglycan recognition protein 1 |
| CTSB = Cathepsin B | PKM = Pyruvate kinase |
| DEFA3/DEFA1* = Defensin, alpha 3 / alpha1 | RPS6* = Ribosomal protein S6 |
| DSG1* = Desmoglein-1 | RPS7* = Ribosomal protein S7 |
| DSP = Desmoplakin | RPSA* = Ribosomal protein SA |
| EEF1D* = Elongation factor 1-delta | RPS18* = Ribosomal protein S18 |
| EEF2* = Eukaryotic elongation factor 2 | RPS19* = Ribosomal protein S19 |
| FGG = Fibrinogen gamma chain | RPS21* = Ribosomal protein S21 |
| FLNA = Filamin A | S100A8* = S100 calcium-binding protein A8 |
| FLNC* = Filamin C | TPM3* = Tropomyosin 3 |
| H2AFY = Core histone macro-H2A | VCL* = Vinculin |
| HIST1H2AJ/HIST1H2AH/H2AFJ/HIST2H2AC/  HIST1H2AC/HIST3H2A/HIST2H2AA3/  HIST1H2AD/HIST1H2AG/HIST1H2AB*= Histones | WISP2* = WNT1-inducible-signaling pathway protein 2 |
| HSPA8 = Heat shock protein family A member 8 | |
| The proportion of 0-values ≥40% in proteins indicated with an asterix (*) | |

| **Table S7.** Table of odds ratios, 95% confidence intervals and p-values for non-significant microdialysate-DEPs for DVT at 2 weeks. | | | | | | |
| --- | --- | --- | --- | --- | --- | --- |
|  |  | DVT at 2 weeks ^n=28^ | | | | |
|  |  | **OR (95% CI)** | ***p*** |  | **OR (95% CI)** | ***p*** |
| ACTA* |  | 4.9 (0.93-26.0) | *0.061* | IGFBP3* | 2.5 (0.52-12.0) | *0.250* |
| ARG1* |  | 3.9 (0.76-20.0) | 0.102 | KPRP* | 4.9 (0.93-26.0) | *0.061* |
| B2M |  | 4.9 (0.93-26.0) | *0.061* | KRT78* | 4.9 (0.93-26.0) | *0.061* |
| BASP1* |  | 2.5 (0.52-12.0) | *0.250* | LGALS3* | 2.5 (0.52-12.0) | *0.250* |
| CAP1 |  | 4.9 (0.93-26.0) | *0.061* | LYZ | 4.9 (0.93-26.0) | *0.061* |
| CLU |  | 4.9 (0.93-26.0) | *0.061* | PCOLCE | 4.9 (0.93-26.0) | *0.061* |
| COL15A1* |  | 2.5 (0.52-12.0) | *0.250* | PGLYRP1* | 4.9 (0.93-26.0) | *0.061* |
| COL18A1 |  | 4.9 (0.93-26.0) | *0.061* | PKM | 4.9 (0.93-26.0) | *0.061* |
| COL6A3 |  | 4.9 (0.93-26.0) | *0.061* | RPS6* | 4.9 (0.93-26.0) | *0.061* |
| COMP |  | 4.9 (0.93-26.0) | *0.061* | RPS7* | 4.9 (0.93-26.0) | *0.061* |
| CTSB |  | 1.4 (0.30-6.2) | *0.699* | RPSA* | 4.9 (0.93-26.0) | *0.061* |
| FLNA |  | 4.9 (0.93-26.0) | *0.061* | RPS18* | 4.2 (0.84-21.0) | *0.081* |
| FLNC* |  | 3.9 (0.76-20.0) | *0.102* | RPS19* | 4.9 (0.93-26.0) | *0.061* |
| HIST* |  | 4.9 (0.93-26.0) | *0.061* | S100A8* | 4.9 (0.93-26.0) | *0.061* |
| HSPA8 |  | 4.9 (0.93-26.0) | *0.061* | TPM3* | 2.5 (0.52-12.0) | *0.250* |
| HSPB1* |  | 4.9 (0.93-26.0) | *0.061* | VCL* | 4.9 (0.93-26.0) | *0.061* |
| Logistic regression adjusted for age. Protein expression dichotomized by median. The proportion of 0-values ≥40% in proteins indicated with an asterix (*) Abbreviations: ACTA = ACTA1/ACTC1/ACTA2/ACTG2. ATRS=Achilles tendon Total Rupture Score, DEP = differentially expressed proteins, DVT= Deep Venous Thrombosis, DEP = differentially expressed proteins, OR= Odds ratio. 95% C. I.= 95% confidence interval for the odds ratio. ACTA= Actins, ARG1= Arginase 1, B2M= Beta-2-mikroglobulin, BASP1= Brain acid soluble protein 1, CAP1= Adenylyl cyclase-associated protein 1, CLU=Clusterin, COL15A1= Collagen, type XV, alpha 1, COL18A1= Collagen, type XVIII, alpha 1, COL6A3= Collagen, type VI, alpha 3, COMP= Cartilage oligomeric matrix protein, CTSB= Cathepsin B, FLNA= Filamin A, FLNC= Filamin C, HIST= Histones, HSPA8= Heat shock protein family A member 8, HSPB1= Heat shock protein family B member 1, IGFBP3= Insulin-like growth factor-binding protein 3, KPRP= Keratinocyte proline rich protein, KRT78= Keratin, type II cytoskeletal 78, LGALS3=Galectin-3, LYZ=Lysozyme, PCOLCE= Procollagen C-endopeptidase enhancer, PGLYRP1= Peptidoglycan recognition protein 1, PKM= Pyruvate kinase, RPS6= Ribosomal protein S6, RPS7= Ribosomal protein S7, RPSA= Ribosomal protein SA, RPS18= Ribosomal protein S18, RPS19= Ribosomal protein S19, S100A8= S100 calcium-binding protein A8, VCL= Vinculin | | | | | | |
